# The Interaction of *mTOR* and *Nrf2* in Neurogenesis and Its Implication in Neurodegenerative Diseases

**DOI:** 10.3390/cells11132048

**Published:** 2022-06-28

**Authors:** Linda Ines Zoungrana, Meredith Krause-Hauch, Hao Wang, Mohammad Kasim Fatmi, Lauryn Bates, Zehui Li, Parth Kulkarni, Di Ren, Ji Li

**Affiliations:** 1 Department of Surgery, Morsani College of Medicine, University of South Florida, Tampa, FL 33612, USA; lindainesz@usf.edu (L.I.Z.); meredithk@usf.edu (M.K.-H.); wangh@usf.edu (H.W.); fatmi@usf.edu (M.K.F.); lbates2@usf.edu (L.B.); diren@usf.edu (D.R.); 2 Department of Medical Engineering, College of Engineering and Morsani College of Medicine, University of South Florida, Tampa, FL 33612, USA; zehui@usf.edu (Z.L.); parth12@usf.edu (P.K.)

**Keywords:** *mTOR*, *Nrf2*, neurogenesis, neurodegenerative diseases

## Abstract

Neurogenesis occurs in the brain during embryonic development and throughout adulthood. Neurogenesis occurs in the hippocampus and under normal conditions and persists in two regions of the brain—the subgranular zone (SGZ) in the dentate gyrus of the hippocampus and the subventricular zone (SVZ) of the lateral ventricles. As the critical role in neurogenesis, the neural stem cells have the capacity to differentiate into various cells and to self-renew. This process is controlled through different methods. The mammalian target of rapamycin (*mTOR*) controls cellular growth, cell proliferation, apoptosis, and autophagy. The transcription factor *Nrf2* (nuclear factor erythroid 2-related factor 2) is a major regulator of metabolism, protein quality control, and antioxidative defense, and is linked to neurogenesis. However, dysregulation in neurogenesis, *mTOR*, and *Nrf2* activity have all been associated with neurodegenerative diseases such as Alzheimer’s, Huntington’s, and Parkinson’s. Understanding the role of these complexes in both neurogenesis and neurodegenerative disease could be necessary to develop future therapies. Here, we review both *mTOR* and *Nrf2* complexes, their crosstalk and role in neurogenesis, and their implication in neurodegenerative diseases.

## 1. Introduction

Neurogenesis is a process that gives rise to new neuronal cells from neural stem cells (NSCs) in the brain during embryonic development and throughout adulthood [1]. NSCs can differentiate into different cells and have the ability for self-renewal [1,2]. Under normal physiological conditions, neurogenesis occurs in the hippocampus [2]. Adult neurogenesis persists in two regions of the brain—the subgranular zone (SGZ) in the dentate gyrus of the hippocampus and the subventricular zone (SVZ) of the lateral ventricles [1,2,3,4]. Cells are generated in the SGZ granule and play a critical role in learning and memory. Conversely, SVZ astrocyte-like cells produce transit-amplifying cells that will then produce neuroblasts [3,5]. This then migrates to the rostral migratory stream of the olfactory bulb [3,5,6,7]. NSCs must be able to differentiate, divide, and migrate in order to function properly. In adults, the process of neurogenesis is stimulated by a variety of physiological, pathological, and pharmacological stimuli. An overproduction or alternation of these stimuli has been seen in neurodegenerative diseases. For instance, genes such as *tau*, *presenilin (PSEN)1*, and *huntingtin* are physiologically involved in modulating brain plasticity in an embryonic brain. Studies have shown that *tau* pathology is correlated to functional deficits in Alzheimer’s disease (AD). Mutations in the *huntingtin* gene can lead to the development of Huntington’s disease, and changes in *PSEN1* expression can also cause neurodegeneration and dementia in familial Alzheimer’s disease [8,9,10,11]. Additionally, alterations in the dentate gyrus of the hippocampus, SVZ, and olfactory bulb have been observed in diseases such as Parkinson’s disease (PD) and Huntington’s disease (HD) [11]. This further enhances the question of the link between neurogenesis and the development of neurodegenerative disease.

Many cellular activities such as proliferation, cell growth, and protein synthesis are regulated by the mammalian target of the rapamycin (*mTOR*) pathway, a key regulator of these processes [12]. Upon activation, the *mTOR* complex is phosphorylated through two of its substrates—the eukaryotic initiation factor 4E (eIF4E)-binding proteins 4E-BP1 and 4E-BP2 and the ribosomal S6 kinases S6K1 and S6K 2 [13]. Several studies have shown the involvement of *mTOR* in neurogenesis as its activation can change NSC migration, differentiation, maturation, and dendrite development [14]. Furthermore, elevated phosphorylated *mTOR* and its downstream protein targets have been observed in AD pathology suggesting the implication of *mTOR* in neurodegenerative diseases [15,16,17]. An increase in oxidative stress is one of the key mechanisms leading to the development of AD [18]. This stress is known to interfere with neural survival proliferation and differentiation, suggesting nuclear factor erythroid 2-related factor (*Nrf2*) could induce NSC proliferation in the subgranular zone of the dentate gyrus during oxidative stress [18]. Neuronal cells differ from any other cells in shape, function, mode of activation, and renewal. For instance, endothelial cells such as skin cells can function independently, while neuron cells depend on the stimulation and neurotransmitters generated by other nerves to function. Nerve cells consist prominently of the nucleus, and once these cells are destroyed, new cells cannot be generated. Due to those variations, the key mechanism of activation between *mTOR* and *Nrf2* might have differences.

After years of investigating neurodegenerative diseases such as AD, PD, and HD, researchers still have not found the best treatment methods. Studies have still not been fully able to answer the questions of how protein folding, and neuronal cell death are associated with AD, PD, and HD. Further, can neuronal replacement be used as a form of therapy? Can molecular mechanisms associated with neurodegenerative diseases and neurogenesis be used as a treatment target? All of these questions are still not answered. Moreover, even after decades of investigations in neurogenesis and the field of NSC by neurobiologists, there are questions that remain unclear, such as can the mechanism and factors that regulated this process can be used to treat neurological disease? To what extent is neurogenesis beneficial since having more new neurons is not always better [19]? Furthermore, in pathological and physiological states, what are the molecular and cellular mechanisms present within the brain that would lead to the repair or reshaping of the neurons?

As regenerative medicine, also known as stem cell therapy, opens a new direction in modern medicine, understanding key elements associated with both neurogenesis and neurodegenerative diseases could provide new insight into certain neurodegenerative treatment methods. In this review, we discuss *mTOR* and *Nrf2* complexes, their crosstalk, and roles in neurogenesis, and the implication in neurodegenerative diseases such as AD, PD, and HD, and how a better understanding of those complexes could provide possible answers to questions mentioned previously. Our objective is to provide an overview of the implication of *mTOR* and *Nrf2* in neurogenesis as the formation of neuronal cells and neuronal cell death play a critical role in embryonic and adult brain development. Further changes in these processes have been seen in neurodegenerative diseases, which we further evaluate in this review.

## 2. Nrf2 in Neurogenesis and Disease Development

### 2.1. Nrf2 Mechanism Pathway

**Figure 1:**
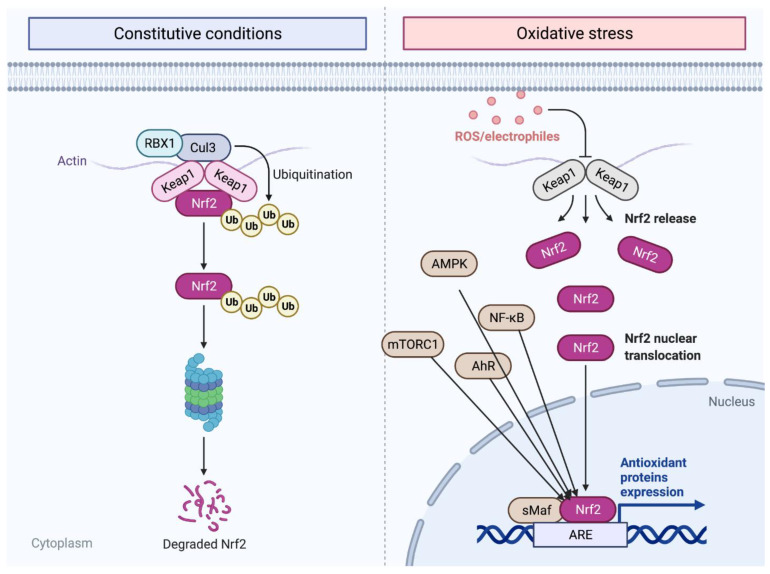
*Nrf2* mechanism pathway. *Nrf2* is released in the cytoplasm then migrates into the nucleus where it is transcripted during oxidative stress and degraded when it is no longer needed through ubiquitination (see text for more information). The figure was prepared by software provided by Biorender.com (accessed 3 March 2022).

One of the key regulators of cellular oxidation is nuclear factor erythroid 2-related factor 2 (*Nrf2*) which is a key transcriptional factor that regulates different genes in cells under both normal and stress conditions [20,21]. *Nrf2* maintains cellular homeostasis by activating the expression of cytoprotective, antioxidant, and anti-inflammatory genes [21]. *Nrf2* is a basic leucine zipper (bZip) transcription factor from the cap ‘n’ collar (CNC) family which includes p45 NF-E2, NRF1, NRF3, CNC homolog 1 (Bach 1) and Bach 2, broad-complex, tramtrack, and bric-a-brac (BTB) [20,22]. One difference between the members of this family of transcription factors is that *Nrf1* through 3 acts as a transcriptional activator, while Bach 1 and 2 serve as transcriptional repressors, and play a role in regulating *Nrf2* [20]. In the nucleus, *Nrf2* can heterodimerize with small MAF or JUN proteins. When *Nrf2* heterodimerizes with small MAF (sMaf), it enhances nucleotide sequences in the DNA called antioxidant response elements (*ARE*) or electrophile response elements (EpRE) [20,21]. Brain-derived neurotrophic factors show that *Nrf2* cellular function includes metabolic regulation, protein quality control, and antioxidative defense by initiating the transcription of cytoprotective genes, including anti-inflammatory interleukin (IL)-10, peroxisome proliferator-activated receptor gamma coactivator 1-alpha (PGC-1α), and iron exporter ferroportin 1 [20,21]. The *Nrf2* molecular structure consists of seven *Nrf2*-erythroid-derived CNC homology (ECH; Neh) domains (Neh1-7), that are critical in *Nrf2* repression and activity [20]. For instance, Kelch-like ECH associated protein 1 (Keap1) is a negative regulator of *Nrf2* under unstressed cellular conditions [20]. Moreover, Neh6 is important for *Nrf2* degradation in stressed cells independent of Keap1 while on the other hand Neh3 interacts with other transcription factors through DNA binding and dimerization [20]. *Nrf2* is regulated in both the nucleus and the cytosol [20]. Glycogen synthase kinase 3 (GSK3) mediated proteasomal degradation of *Nrf2* in the nucleus while Keap1-mediated proteasomal degradation of *Nrf2* occurs in the cytosol with RBX1 and Cul3 [20]. *Nrf2* transcription factor is activated by Aryl hydrocarbon receptor (AhR), nuclear factor (NF)-κB (NF-κB), p53, myocyte-specific enhancer factor 2 D (MEF2D), breast cancer 1 (BRCA1), proliferator-activated receptor (PPAR)α or PPARγ, c-Jun, and c-Myc. All can activate *Nrf2* transcription, while the mechanistic target of rapamycin complex 1 (mTORC1), AMP-activated protein kinase (AMPK), as well as GSK3, all play a role in *Nrf2* stability and activity by directly or indirectly interacting with *Nrf2* [20]. With this interconnection of *Nrf2* with other genes and proteins, it is no surprise that *Nrf2* might be linked to neurogenesis and the development of neurodegenerative diseases. A summary of the *Nrf2* signaling pathways is presented in Figure 1. Although the *NRF2* pathway plays a protective role against regulating oxygen reactive species (ROS), some studies have suggested that both the suppression of the activity of the *Nrf2* transcription factor and priming are necessary for the upregulation of antioxidant molecules. However, there are contradictions among the literature regarding cell-generated memory mechanisms of activation [23,24].

### 2.2. Nrf2 in Neurogenesis

As previously mentioned, *Nrf2* controls cellular homeostasis linked to multiple stressors by regulating other genes involved in the anti-inflammatory response, metabolic reprogramming of tumor cells, antioxidant defense, autophagy, etc. [20,21]. The expression of those genes promote self-renewal, cell survival, differentiation, cell growth, proliferation, and increased lifespan [25]; all of which are important factors to the function of stem cells [25]. Some studies have explored the *Nrf2* association in neurogenesis and found that the decline in *Nrf2* expression during aging was linked to the reduction of neural stem cells in the SVZ [25,26]. Other studies have looked into the role of *Nrf2* in the homeostasis of the neurogenic niche in the SGZ and reported that the overexpression of *Nrf2* enhances neuronal differentiation. They have also found that an upregulation of *Nrf2* improves amyloid β-mediated neural stem cell death, and genetic change in *Nrf2* expression can either rejuvenate or suppress the neural stem cell niche in the SGZ [18,21,25].

One of the main characteristics of Alzheimer’s disease (AD) development is the extracellular deposits of amyloid β (Aβ) and the increase in oxidative stress associated with Aβ toxicity; so understanding the mechanism of *Nrf2* in this process could lead to a new therapeutic approach in AD [18,27]. Regulation of oxidative stress also has an impact on neural stem cell differentiation. *Nrf2* has an impact on neural stem cell survival, differentiation, and neurogenesis by ROS [21]. ROS, which is produced in the endoplasmic reticulum (ER), membrane-bound NADPH oxidase, and cellular mitochondria, is the main oxidative stressor that is constantly produced by cells during metabolic functions [21,28]. The amounts of ROS produced has been associated with cellular survival, proliferation, and differentiation [21,28]. The overproduction of ROS in the mitochondria of neural stem cell in the presence of glucose alters endogenous antioxidant homeostasis leading to oxidative stress [21,29]. A study of neural stem cell lines (i.e., C17.2) shows that high glucose-mediated oxidative stress induces endoplasmic reticulum (ER) stress, inhibiting C17.2 cell differentiation into glia or neuron cells [21,29]. Modulating physiological ROS signaling and stimulating *Nrf2*-dependent developmental genes, in primarily the mitochondria as well as the ER, determines the molecular metabolism and the fate of NSC is crucial [21,30]. Studies conducted on the *Nrf2* knockout rate in different ages show that *Nrf2* expression regulates the glial differentiation of NSC in the dentate gyrus as well as neurogenesis-related hippocampal behaviors [21,31]. Semkova et al. presented in their study that both *Nrf2* proteasomal activity and pathway response to oxidative stress was crucial to developing the nervous system, showing the importance of *Nrf2* in neurogenesis [32]. However, the stress that could be encountered during neurogenesis has not been fully explored, which can present limitations on *Nrf2* mechanistic pathways associated with neurogenesis. *Nrf2* is an important factor in NSC proliferation, differentiation, and survival, and could provide a further understanding of the fundamental aspects of NSC biology.

### 2.3. Nrf2 in AD

AD is one of the most common neurodegenerative diseases and its medical management is still a challenge [33]. AD pathology is associated with an aggregate of Aβ plaques as well as intracellular aggregations of neurofibrillary tangles (NFTs), composed of hyperphosphorylated microtubule-associated τ and p-tau [34]. Aβ plaques develop and spread from the basal neocortex regions of the brain to the hippocampus, amygdala, and basal ganglia [34]. In patients with advanced AD, Aβ was found further in the cerebellar cortex and throughout the mesencephalon, while in the critical stage, it spread to the hippocampus and neocortex [34]. The abnormal concentration of Aβ causes a τ-tangle to form, which is mostly found in the locus coeruleus and transentorhinal and entorhinal are as of the brain [34].

In the aged population, *Nrf2* expression decreases. This phenomenon is also observed in AD patients [35]. The correlation between low *Nrf2* and AD can be explained due to *Nrf2* playing a role in inflammation, oxidative stress, and influencing autophagy directly or indirectly [36,37,38]. Rojo et al. (2017) and Joshi et al. (2015) demonstrated a decline in *Nrf2* in AD animal models and aggregate-like pathology enhancing *Nrf2* correlation to AD [38,39]. Some of the *Nrf2* target genes, Heme oxygenase-1 (HO-1), NADPH quinone oxidoreductase I (NQO1), and glutamate-cysteine ligase catalytic subunit (GCLC) have been observed in AD brains [37,40]. For instance, NQO1, which protects the plasma membrane from free radicals and lipid peroxidation, is upregulated in the AD frontal cortex region [37]. Bahn et al. (2019) in their study showed that high expression of NQO1 expression in 3xTg-AD mice leads to Aβ immunoreactivity [37,41]. Furthermore, Rojo et al. (2017) observed an increase in Aβ and p-tau expression in *Nrf2*-deficient mice [39]. Other studies implicate the link between *Nrf2* and chaperone-mediated autophagy [37]. Pajares et al. (2018) identified *Nrf2* binding sequences in lysosomal-associated membrane protein 2A (*LAMP2*) in both mouse models and humans. Moreover, an overexpression and under expression of *Nrf2* is linked to an increase or reduction in *LAMP2* [37,42]. *Nrf2* deletion has been associated with an increase in intracellular Aβ42 and Aβ40 [37,38]. Bahn et al.’s study of beta-site amyloid precursor protein cleaving enzyme 1 (BACE1) revealed that Nrf2 inhibits BACE1, a rate-limiting enzyme for Aβ peptides in AD model mouse, by binding to the are promoter of BACE1 [37,41]. Finally, Nrf2 affects p-tau by inducing nuclear dot protein 52 (NDP52), an autophagy-associated protein linked in p-tau degradation and binding in its are promoter [37,43]. This suggests that *Nrf2* could facilitate tau clearance [37,43]. Further study of the regulatory mechanism of *Nrf2* in AD could provide a better understanding of the disease development.

### 2.4. Nrf2 in PD

PD is one of the most common neurodegenerative diseases that affect individual movements. The pathogenesis of PD is characterized by the degeneration of dopamine neurons in the midbrain leading to the loss of dopamine neurotransmitters, the primary motor neurons [37,44]. This results in symptoms such as ataxia, bradykinesia, and rigidity [44]. In addition to the loss of dopamine, the presence of protein inclusions such as Lewy bodies have been observed in PD [44]. Further, the upregulation of free radical-generating enzymes is another attribute that has been observed in PD [37,45]. Guo et al. observed that *Nrf2* activity was reduced in the 1-methyl-4-phenyl-1,2,3,6-tetrahydropyridine (MPTP) model of PD, which enhances the PD phenotype and MPTP has been associated with iron deposit and astroglia HO-1 expression [37,46,47]. Furthermore, studies have shown that NQO1 expression in PD links *Nrf2* to its neuroprotective effect [37,48]. An increase in proinflammatory cytokines released by activated microglia was observed in the cerebrospinal fluid of PD patients [37,49]. Another study by Rojo et al. has shown that *Nrf2* decreases microglial activation in PD progression [50].

Moreover, studies have shown that the PI3K/AKT/GSK3β signaling axis is involved in PD neuroprotection, and a decline in *AKT* is associated with sporadic PD [37]. In addition, elevated expression of GSK3β expression and activity has been reported in PD, and inhibition of GSK3β is known to increase antioxidant genes as well as *Nrf2* activity [37]. In summary, *Nrf2* has potential neuroprotective effects in PD.

### 2.5. Nrf2 in HD

HD is a rare hereditary neuronal disease contrary to AD and PD, which are caused by mutations or protein misfolding. HD autosomal hereditary disease is caused by CAG trinucleotide repeat expansion in the huntingtin (*HTT*) gene leading to an expansion of polyglutamine repeats in the huntingtin protein (mtHtt) [51,52]. This mutation causes progressive degeneration of nerve cells in the brain and leads to a lack of movement coordination along with motor impairments [51]. Even though there is a lot to learn about HD, studies have shown that cellular antioxidants, as well as mitochondrial dysfunction, play a role in HD pathology connecting *Nrf2* to HD [51]. Enhanced lipid peroxidation has been observed in HD mice. Impairment in this antioxidant mechanism can lead to the inhibition of *Nrf2* activity by mtHtt [51]. mtHtt interacts with the CBP/p300 dimer, preventing its acetylation and function, which then blocks *Nrf2* cellular localization and stability [51,53]. The mechanism by which *Nrf2* affects the formation and aggregation of huntingtin protein is not fully understood. However, studies have found that *Nrf2* activation in certain parts of the brain have a neuroprotective effect and extends the lifespan in HD animal models [51,54]. Furthermore, Saito et al. (2020) observed that *Nrf2* induced the expression of p62 autophagy-related proteins and microtubule-associated protein 1A/1B-light chain 3 (LC3) [55]. Both aid in the rapid clearance of toxic of mtHtt aggregates by forming a shell around it [55].

The deposit of toxic mtHtt aggregate, astrocyte, and microglia activation all contribute to HD pathology and progression. This deposit triggers the release of cytokines and pro-inflammatory mediators [51]. The connection between NF-κB and the *Nrf2* pathway is well documented and shows the activated *Nrf2* upregulation of HO-1. This upregulation decreases the expression of pro-inflammatory cytokines and reduces inflammatory mediator levels [51,56,57]. The *Nrf2*/*ARE* pathway assists in the neuroinflammation caused by mtHtt accumulation [51,56,57]. The interaction between NF-κB and the *Nrf2* pathway could be the target of new treatment therapies for HD.

## 3. *mTOR* in Neurogenesis and Disease Development

### 3.1. mTOR Mechanism Pathway

**Figure 2:**
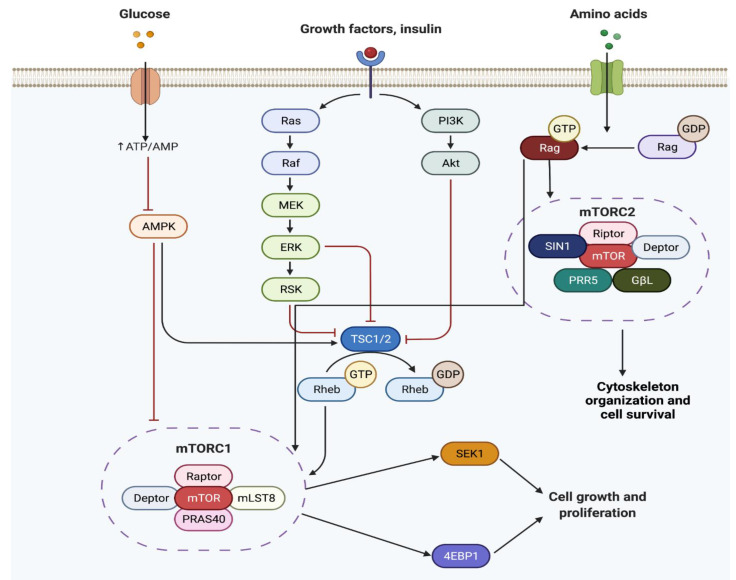
*mTOR* mechanism pathway. Glucose and growth factors activate a series of pathways that either activate or inhibit (AMPK) mTORC1. Amino acids can either activate mTORC1 or mTORC2, which lead to cytoskeleton organization and cell survival or cell growth and proliferation (see text for more details). The figure was prepared by software provided by Biorender.com (accessed on 3 March 2022).

Cellular development such as cell proliferation, autophagy, apoptosis, cellular growth, and more are controlled by the mammalian target of rapamycin (*mTOR*) [58,59]. *mTOR* is a member of the phosphatidylinositol 3-kinase-related kinase family of protein kinases [58,59]. Through the phosphorylation of p70S6K and 4E-BP1 and the regulation of cap-dependent translation, *mTOR* activates protein synthesis [60]. *mTOR* plays an important role in the body and its mutation or dysregulation has been associated with diseases such as cancer, cardiovascular disease, obesity, inflammation, diabetes, and neurodegenerative disease [58,61]. *mTOR* has two functionally and structurally distinct complexes known as the mammalian target of rapamycin complex 1 (mTORC1) and the mammalian target of rapamycin complex 2 (mTORC2) [58] (Figure 2). mTORC1 complex consists of mTOR, GβL, regulatory-associated protein of *mTOR* (raptor), and DEP domain-containing *mTOR* interacting protein (DEPTOR) genes [58,62]. mTORC1 activity is influenced by growth factors, amino acids, energy levels, glucose availability, and stressors [58,62]. mTORC2 complex consists of mTOR, SIN1, Rictor, PRR5, GβL, and DEPTOR genes [58,61]. This complex controls cytoskeleton organization and cell survival as shown in Figure 2 [58,61]. *mTOR* is involved in multiple cellular signaling pathways such as adenosine 5′-monophosphate-activated protein kinase (AMPK), phosphoinositide-3-kinase (PI3K)/AKT, tuberous sclerosis complex subunit 1 and 2 (TSC1 and TSC2), Rag GTPases, Rheb, etc. [56]. Although there is still a lot to learn about mTORC2, such as the type of proteins involved in the activation mechanism or proteins that make up its complex, both downstream and upstream pathways of mTORC1 are better understood. For instance, it is known that mTORC1 activates central nervous system hormones, neuromodulators, and neurotransmitters [61]. The interaction of *mTOR* with its downstream substrate leads to diverse cellular responses associated with neurogenesis.

### 3.2. mTOR in Neurogenesis

The role of the mTORC1 and mTORC2 complex in stem cell maintenance, growth, and differentiation vary due to their downstream and upstream targets. Changes in the upstream elements in mTORC1 signaling can lead to NSC proliferation [14]. For instance, UTX, a histone demethylase, promotes *PTEN* expression, and the deletion of both UTX and *PTEN* during neural development increase AKT and mTOR phosphorylation resulting in an increase in NSC proliferation [63]. When AKT is overexpressed it can induce proliferation in cortical progenitors [14,64]. Cell proliferation is enhanced by *AKT* activation through mTORC1 mechanisms [14,64]. Inhibition of mTORC1 through DEPTOR plays an important role in maintaining stemness, and when the cell is about to differentiate it is downregulated [65]. mTORC1 plays an important role in NSC’s differentiation into daughter cells [14,60]. This differentiation reduces mTORC1 activity, in turn decreasing neural progenitor cell populations [14,60]. Insulin, one of the growth factors that stimulated mTORCs through the activation of AKT, PI3K, Rheb, and the TSC pathway, also promotes neuronal differentiation in NSC through mTORC1 activation [14,61]. Furthermore, activation of mTORC1 after TSC1 deletion decreases the proliferation of stem cells in the SVZ [66]. Dysregulation of mTORC1 can also alter neurite development as well as synapse formation and maintenance resulting in epilepsy [14]. Through the activation of different pathways, *mTOR* is involved in neurogenesis.

### 3.3. mTOR in AD

As mentioned earlier, AD pathology is associated with an aggregate of Aβ plaques, intracellular aggregations of neurofibrillary tangles (NFTs), and composed of hyperphosphorylated microtubule-associated τ and p-tau [34]. The implication of *mTOR* in AD pathogenesis is not fully understood but evidence shows that in the mouse model Aβ production was greatly reduced [67]. AMPK is activated suggesting an indirect implication of *mTOR* in AD [67]. Furthermore, Oddo et al. provided evidence that Aβ aggregated causes hyperactivation of *mTOR* which then hyperphosphorylated *tau*, in turn leading to PHF’s (paired helical filaments) and NFT’s formation [67,68].

The analysis conducted on the human AD brain shows a high level of p-mTOR as well as two of its downstream targets, p70S6K and eIF4E, suggesting a high *mTOR* activity in AD [68]. According to Jin-Jing Pei et al., an increase in p70S6K could be associated with the upregulation of tau in AD [69]. Studies have shown that *mTOR* activation also enhances tau pathology, leading to the dysfunction of autophagy [68]. In summary, *mTOR* plays an important role in both anabolic and catabolic processes that are involved in aging. However, full understanding of the mechanisms remains unclear and requires further investigations.

### 3.4. mTOR in PD

Recent evidence has shown that *mTOR* signaling is altered during PD progression. However, whether *mTOR* has neurotoxicity or neuroprotection remains unclear and controversial. For instance, some studies have shown that 1-methyl-4-phenyl-1,2,3,6-tetrahydropyridine (MPTP) and rotenone 6-hydroxydopamine (6-OHDA) suppress *mTOR* activity [70,71,72,73]. Conversely, Domanskyi et al., found that deletion of *PTEN* led to the activation of *mTOR* and protection of dopaminergic neurons against neurotoxin insult in PD mouse models [73,74].

In addition, REDD1 and mTORC1 inhibitors are upregulated in cellular PD models suggesting that while elevated REDD1 expression suppresses *mTOR* signaling, inhibition of REDD1 has a neuroprotective effect on PD animal models [73]. In other studies, *AKT*/*mTOR* signaling seems to have a regenerative effect in dopaminergic neurons, enhancing the neuroprotective effect of *mTOR* in certain PD models [73,75]. The neuroprotective effect of *mTOR* in PD is unclear and the findings are contradictory on whether *mTOR* has a neurotoxic or neuroprotective effect in PD models.

### 3.5. mTOR in HD

The mechanism of mHtt in HD is still not fully understood. However, since *mTOR* inhibits autophagy, it could be implicated in the pathology by having a protective effect in HD. Nevertheless, mTORC1 promotes many mechanisms that are impaired by mHtt [76]. In a study conducted by Lee et al. (2015), the phosphorylation of ribosomal protein S6, a marker of mTORC1 activity, was decreased in HD patients [77]. As presented in Figure 2, the mutation of mTORC1 activators, Rheb and caRheb, in mice models increased elements that promote mitochondrial function, autophagy, and cholesterol synthesis, which result in a depletion of HD [77]. Furthermore, mHTT aggregate abundance and expression of genes that promote mHtt aggregation is decreased, improving motor performance and decreased atrophy [76,77].

However, mTORC1 is predominantly regulated by Ras homolog enriched in the striatum (Rhes). A large decrease of Rhes occurs in HD patients. This suggests that the activation of mTORC1 may be beneficial in HD [76,77]. Furthermore, *mTOR* activity contributes to the brain atrophy and neuronal shrinkage observed in HD [78,79]. When *mTOR* targets S6K1, the phosphorylation and activity of mHtt is reduced [78,79]. Further investigation of the mechanism of *mTOR* activity in HD is required.

## 4. *mTOR* and Nrf2 Crosslink Signaling Pathways

**Figure 3:**
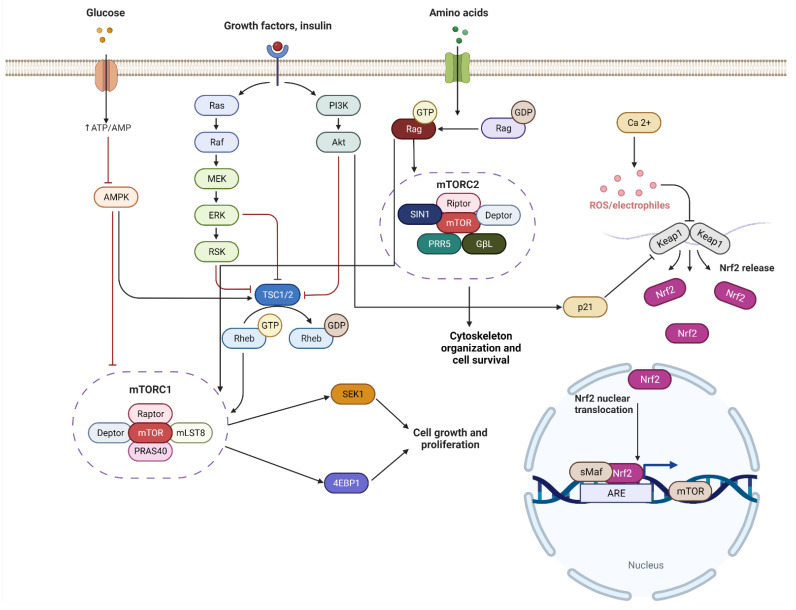
*Nrf2* and *mTOR* mechanism pathway. *AKT* is one of the procedures of *mTOR* activity, which plays an important role in cellular mechanisms such as cell proliferation, cell cycle, and activated p21, which then inhibits Keap1, which alternates the *Nrf2* mechanism. The figure was prepared by software provided by Biorender.com (accessed 3 March 2022).

*Nrf2*/*ARE* signaling is associated with the *AKT*/*PI3K* pathway and the activation of both protects against oxidative stress [80]. *Nrf2*/*ARE* positively regulates *mTOR* through its interaction with RagD, small G-proteins, which activate mTORC1 [80,81,82]. In glioma cells, downregulation of the *Nrf2* expression by shRNA suppresses *mTOR* while causing the ATP deficit to activate AMPK, leading to *Nrf2* inhibition of *mTOR* [80,83]. One of the most obvious connections between *Nrf2* and *mTOR* is the ARE sequence in the *mTOR* promoter region linking *Nrf2*, which has a direct role in regulating the expression of this gene. This regulation depends on the activity of *PI3K*/*AKT* [80] (Figure 3). The close involvement of the *Nrf2*/*ARE* signaling pathway in *mTOR* function and its association with neurogenesis and neurodegenerative diseases can open new prospective treatments for numerous neurodegenerative diseases. While the crosstalk between *mTOR* and *Nrf2* in neurodegenerative diseases could provide novel therapeutic treatments, there are limited studies that have investigated the mechanism. In the current research available, we were able to find a possible link between memory formation and AD development. Long-term memory formation depends largely on synapse shape, formed by CA1 pyramidal neurons, which depends on N-methyl-d-aspartate glutamate receptors (NMDARs) and Schaffer collateral [84]. During long-term memory formation, an increase in postsynaptic membranes and sensitivity to neurotransmitters are required [80,85]. Through protein synthesis, mTORC1 activation plays an important role. It is involved in the regulation of proteins through cap-dependent mRNA translation of multiple proteins that participate in long-term memory formation [80,85]. To investigate the effect of *Nrf2* on long-term memory, Zweig et al. (2020) studied *Nrf2*^−/−^ mice that exhibited memory impairment and found a decrease in synaptic density and dendritic arborization [80,86]. In AD development, *Nrf2* and *mTOR* function is disturbed and as presented, both play a role in synapse shape associated with memory formation, which suggested their involvement in memory depletion in AD.

## 5. Conclusions and Recommendations for the Future

Neurogenesis occurs in the brain during embryonic development and throughout adulthood. Even during stress conditions or disease, new neuronal cells are produced in the subgranular zone in the dentate gyrus of the hippocampus, and the subventricular zone of the lateral ventricles. However, during disease development, when certain factors occur such as an alteration in *Nrf2* and *mTOR* function, it can lead to the increase or decrease in the activity of progenitor cells. This enhances disease development (i.e., Alzheimer’s disease). Throughout the development of the nervous system, *Nrf2* and *mTOR* play an important role, whether it is directly or indirectly through the activation of other signaling pathways in the differentiation, proliferation, self-renewal of NSC, or in synaptic formation. They are also essential to neurogenesis and the establishment of neural circuits. Changes in their activity have been associated with a few neurodegenerative diseases. *mTOR* and *Nrf2* complexes are associated with external signals and transduction pathways and understanding the role of these complexes are necessary to develop future therapies for current neurodevelopmental disorders.

We recommend further investigations into *mTOR* and *Nrf2* crosstalk in neurogenesis. As we have presented in this review, a better understanding of their interaction could lead to the development of therapeutic drug treatments against neurodegenerative diseases. Although we have a general understanding of the mechanism of *Nrf2* and *mTOR* in some cell types, there are still limitations to our knowledge of their mechanisms in neuronal cells. Further studies using mouse models and investigation into the upregulation or downregulation of various protein activity could provide a better understanding of the pathways, such as activated protein C (APC)-*AKT* signaling pathway and APC-AMPK signaling pathway [87,88].

## Data Availability

Not applicable.
